# Thymol Protects Channel Catfish from *Aeromonas hydrophila* Infection by Inhibiting Aerolysin Expression and Biofilm Formation

**DOI:** 10.3390/microorganisms8050636

**Published:** 2020-04-27

**Authors:** Jing Dong, Lushan Zhang, Yongtao Liu, Ning Xu, Shun Zhou, Qiuhong Yang, Yibin Yang, Xiaohui Ai

**Affiliations:** 1 Yangtze River Fisheries Research Institute, Chinese Academy of Fishery Sciences, Wuhan 430223, China; dongjing@yfi.ac.cn (J.D.); zhanglushan1993@163.com (L.Z.); jdongletter@126.com (Y.L.); ningxuxuning@163.com (N.X.); zhoushun@yfi.ac.cn (S.Z.); yangqh@yfi.ac.cn (Q.Y.); yangybin1985@163.com (Y.Y.); 2 Key Laboratory of Control of Quality and Safety for Aquatic Products, Ministry of Agriculture, Beijing 100071, China

**Keywords:** *Aeromonas hydrophila*, aerolysin, thymol, anti-virulence, biofilm

## Abstract

*Aeromonas hydrophila* is an opportunistic pathogen responsible for a number of diseases in freshwater farming. Moreover, the bacterium has been identified as a zoonotic pathogen that threatens human health. Antibiotics are widely used for treatments of infectious diseases in aquaculture. However, the abuse of antibiotics has led to the emergence of antimicrobial resistant strains. Thus, novel strategies are required against resistant *A. hydrophila* strains. The quorum sensing (QS) system, involved in virulence factor production and biofilm formation, is a promising target in identifying novel drugs against *A. hydrophila* infections. In this study, we found that thymol, at sub-inhibitory concentrations, could significantly reduce the production of aerolysin and biofilm formation by inhibiting the transcription of genes *aerA*, *ahyI*, and *ahyR.* These results indicate that thymol inhibits the quorum sensing system. The protective effects of thymol against *A. hydrophila* mediated cell injury were determined by live/dead assay and lactate dehydrogenase (LDH) release assay. Moreover, the in vivo study showed that thymol could significantly decrease the mortality of channel catfish infected with *A. hydrophila*. Taken together, these findings demonstrate that thymol could be chosen as a phytotherapeutic candidate for inhibiting quorum sensing system-mediated aerolysin production and biofilm formation in *A. hydrophila*.

## 1. Introduction

Fish and fishery products provide protein-rich food for human consumption, thus, the aquaculture industry has developed rapidly during the past two decades [1]. However, the increasing growth of freshwater aquaculture has resulted in a number of challenges to successful farming [2]. Among these problems, infections caused by pathogenic *Aeromonas hydrophila* (*A. hydrophila*) have been regarded as a major concern to freshwater fish aquaculture, often leading to severe losses globally [1]. In addition, *A. hydrophila* is an opportunistic pathogen to human and terrestrial animals that can cause a range of infections, such as diarrhea, septicemia, and meningitis [3]. Infections caused by *A. hydrophila* can transmit from diseased fish to humans by intake of contaminated water and undercooked fish [4]. Therefore, *A. hydrophila* has been recognized as a food-borne pathogen by the US Food and Drug Administration since 1984 [5]. Antibiotics and vaccines are the two main approaches to fighting against bacterial infections. Chemotherapy by antibiotics against infections caused by bacterial pathogens leads to the emergence and spread of antibiotic resistance. Although several vaccines have been approved by the national veterinary drug certificate agency in China, these products have not been achieved real industrialized use due to a number of limits [6]. Thus, alternative strategies are urgently needed for combating resistant *A. hydrophila*.

The ever-growing threat of antibiotic resistance has fostered demand for alternative anti-microbial strategies [7], thus leading to anti-virulence strategies. This focus of this study is the identification of novel drugs against *A. hydrophila* from natural compounds based on an anti-virulence strategy. Aerolysin, the 54-kDa pore-forming toxin secreted as proaerolysin, has been considered as a key virulence factor in the pathogenicity of *A. hydrophila* [8]. Proaerolysin is secreted with a flexible 43-residue loop in the C-terminus. Toxin activities will release proaerolysin by cleaving the residues in the C-terminus by trypsin or furin [9]. The toxin exhibits hemolytic, cytotoxic, and enterotoxic activities by forming heptamer with β-barrel pores on target cells [8]. It has been reported that aerolysin can cause the death of a number of cells [10]. Moreover, a previous study demonstrated that the lethal dose of recombinant aerolysin to the channel catfish (average weight = 5.6 ± 0.6 g) was 2 μg per fish by intraperitoneal injection [11]. Moreover, studies have demonstrated that strains lacking the *aerA* gene will decrease the pathogenesis of *A. hydrophila* in animal models [12]. Consequently, aerolysin is a promising target in identifying drugs based on an anti-virulence strategy.

**Figure 1:**
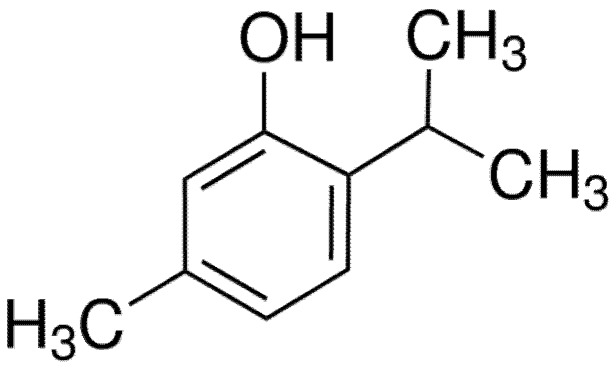
Chemical structure of thymol.

Thymol (Figure 1), belonging to the monoterpene phenol compound, can be extracted from the Lamiaceae family plants, such as the genera *Thymus*, *Ocimum*, *Origanum*, *Satureja*, *Thymbra*, and *Monarda* [13]. Thymol exhibits a variety of pharmacological activities, including antimicrobial, antioxidant, anti-cancerous, and anti-inflammatory, and has been widely used in medicine [14]. In this study, we found that thymol could significantly reduce the expression of aerolysin and the formation of biofilm of *A. hydrophila* at sub-inhibitory concentrations. Moreover, thymol could provide a significant protection against *A. hydrophila* infection in a channel catfish model.

## 2. Materials and Methods

### 2.1. Microorganism and Reagents

*Aeromonas hydrophila* strain XS-91-4-1 (isolated from Silver carp) was provided by Prof. Aihua Li at the Institute of Hydrobiology, Chinese Academy of Sciences. Thymol (purity > 98%) was obtained from the National Institute for Food and Drug Control (Beijing, China). Thymol and enrofloxacin were dissolved in dimethyl sulfoxide (DMSO, Sigma-Aldrich, St. Louis, MO) for preparation of stock solutions at concentrations of 40,960 and 10,240 μg/mL, respectively. For in vivo study, thymol was dissolved in 10% Tween-80 to obtain a thymol emulsion.

### 2.2. Determination of Minimal Inhibitory Concentrations

The broth-dilution method was employed to determine the minimal inhibitory concentrations (MICs) of thymol and enrofloxacin against *A. hydrophila* XS-91-4-1 according to the guidelines of the Clinical and Laboratory Standards Institute (CLSI) [15]. Briefly, the assays were carried out in 96-well plates. Drugs at concentrations ranging from 2 μg/mL to 512 μg/mL for thymol and from 0.125 μg/mL to 32 μg/mL for enrofloxacin were serial 2-fold diluted by MHB medium in a 96-well plate, then bacteria at concentration of about 5 × 10^5^ CFU/mL were added into each well. Following incubation for 18–20 h at 28 °C, the MICs were read by the lowest concentration with no visible *A. hydrophila* growth.

### 2.3. Growth Curves

A volume of 100 mL *A*. *hydrophila* XS-91-4-1 cultures in brain-heart infusion (BHI) medium was aliquoted into a 250 mL flask when the optical density (OD) at 600 nm reached 0.3. Following addition of indicated concentrations of thymol or DMSO (which served as the drug-free group), the mixtures were further incubated for 5 h at 28 °C. The values of OD_600 nm_ were monitored by a spectrophotometer to evaluate the impact of thymol on bacterial growth.

### 2.4. Hemolytic Activity Assay

*A*. *hydrophila* XS-91-4-1 was co-cultured with indicated concentrations of thymol in BHI medium to obtain OD_600 nm_ of 1.5. Then, the cultures were harvested by centrifugation (8000 *g*, 4 °C, 1 min) and supernatants were acquired. Before hemolytic assays, the supernatants were treated with 10 μg/mL trypsin for 10 min at room temperature to activate aerolysin [16]. The reaction system was mixed with 100 μL trypsin-treated supernatant, 875 μL phosphate-buffered saline (PBS), and 25 μL defibrinated sheep red blood cells, then the mixtures were further incubated at 37 °C for 20 min. Unlyzed erythrocytes cells were removed by centrifugation (8000 *g* at room temperature for 1 min). The hemolytic activities of supernatants treated with different concentrations of thymol were determined by measuring the absorption at 543 nm. Sheep erythrocytes treated with Triton X-100 were employed as the positive control.

### 2.5. Western Blot Analysis

Supernatants for Western blot assays were prepared in the same manner as described for the hemolytic activity assay. Concentrations of total protein in supernatants with different treatments were determined by a BCA Protein Assay Kit (Thermo Fisher Scientific, Waltham, MA, USA) according to the instructions. As equal amount of protein was subjected to sodium dodecyl sulfate (SDS)-polyacrylamide (12%) gel after boiling in Laemmli sample buffer [17]. Then, proteins were transferred onto a polyvinylidene fluoride membrane by a semi-dry transfer cell. After blocking with skim milk and incubating with antibodies, protein levels in the membrane were detected with electro chemi luminescence (ECL) Western blotting detection reagents.

### 2.6. Biofilm Formation Assay

Biofilm formation assay in the presence of thymol was performed in 96-well plates with some modifications as previously described [18]. In brief, XS-91-4-1 was cultured in BHI medium overnight, then the cultures were diluted to 1:20 with fresh medium plus thymol at concentrations of 0, 2, 4, 8, 16 μg/mL. After incubation for 24 h at 37 °C without shaking, the OD_600 nm_ values of each culture were determined to ensure that the bacteria had reached the stationary phase with a similar OD_600 nm_. Then, the planktonic cells were removed by washing the plate twice with PBS. The air-dried plates were stained with 0.5% crystal violet for 30 min. After washing with PBS, the crystal violet in attached bacterial cells was released by addition of 30% glacial acetic acid. The influence of thymol on biofilm formation was assessed by measuring the absorption at OD_570 nm_ for each well of the plates. The group with DMSO only served as the positive control and BHI medium was used as the negative control.

### 2.7. RNA Extraction and Real-Time PCR

For RNA extraction, bacterial cells were collected by centrifugation when absorption of the cultures with thymol reached 1.5. Then, total RNA was isolated by a TIANGEN RNAprep Pure Bacteria Kit (TIANGEN, Beijing, China) according to the manufacturer’s instructions. Extra DNA was digested by DNase I, then RNA was reverse transcribed into cDNA by the PrimeScript RT Master Mix (Takara, Kyoto, Japan). The reaction system was mixed with 4 μL 5× PrimeScript RT Master Mix, 10 μL total RNA (500 ng), and 6 μL RNase Free water. cDNA was acquired by incubation at 37 °C for 15 min. Primer pairs for RT-PCR are listed in Table 1 according to previous studies [3,18]. The PCR reaction was performed in a 25 μL volume system consisting of 12.5 μL 2× SYBR Premix Ex Taq (Takara), 0.5 μL of each primer (10 μM), and 1 μL cDNA on a CFX96 Touch Real-Time PCR Detection System (Bio-Rad, Hercules, CA, USA). The PCR conditions were as follow: 95 °C, 30 s; 95 °C, 5 s; 56 °C, 30 s; 72 °C, 20 s; 35 cycles. All samples were analyzed in triplicate and the *16S rRNA* gene served as the internal control to normalize the expression levels of samples with different treatments.

### 2.8. Cell Viability Assays

A549 cells obtained from the American Tissue Culture Collection (ATCC) were cultured in DMEM supplemented with 10% fetal bovine serum. Cells were seeded into 96-well plates at a density of 1.5 × 10^5^ per well for 16 h at 37 °C with 5% CO_2_. Then, 20 μL bacterial supernatants were loaded onto each well and the plates were further incubated for 2 h. Lactate dehydrogenase (LDH) release assay and live/dead assay were performed to determine the cell viability. For live/dead assay, cell images of stained cells were captured by a fluorescence microscope (Olympus, Tokyo, Japan) after treatment with a LIVE/DEAD Viability/Cytotoxicity Kit. LDH activity of cell supernatants was determined on a microplate reader (Bio-Rad, Hercules, CA, USA).

### 2.9. Channel Catfish Model of A. hydrophila Infection

Animal studies were performed under the guidance (Permit No. 20190306002, 6 March 2019) of the Animal Welfare and Research Ethics Committee at Yangtze River Fisheries Research Institute. All the experimental protocols were approved and supervised by the animal care committee. The channel catfish used for the experiments were raised in our experimental breeding center. For survival assay, 100 μL XS-91-4-1 suspension at a concentration of 1.5 × 10^8^ CFU/mL was injected intraperitoneally. Then, fish were administered with 25 mg/mL thymol 6 h post infection using a gavage needle, and at 12 h intervals for 3 days. Fish in the positive control group were injected with bacterial suspension, while the negative control fish were injected with PBS; both groups were administered with 10% Tween 80 at the same intervals as the thymol-treated group. The number of dead fish was monitored every 24 h for 8 days.

### 2.10. Statistical Analysis

The significance of thymol on hemolytic activity, biofilm formation, gene transcription, and LDH release was analyzed using independent Student’s t-test by SPSS 13.0 statistical software (SPSS Inc., Chicago, IL, USA). Survival rate analysis was carried out by GraphPad Software using the Kaplan–Meier estimates method; the significance between thymol and positive group was analyzed using the log-rank test. A *p*-value <0.05 was considered to be statistically significant.

## 3. Results

### 3.1. Thymol has Little Influence on the Growth of A. hydrophila

**Figure 2:**
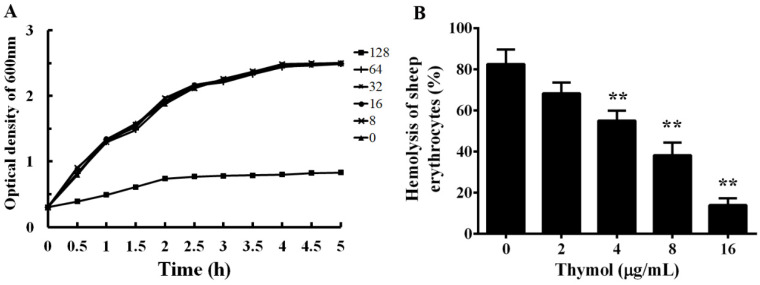
Influence of thymol on the growth and hemolytic activity of *A. hydrophila*. **A**, growth curves of *A. hydrophila* co-cultured with different concentrations of thymol in BHI medium. **B**, Hemolytic activity of *A. hydrophila* supernatants co-cultured with indicated concentrations of thymol; sheep erythrocytes treated with Triton X-100 served as the positive control. All the data in Figure 2B are shown as mean value ± SD of three independent assays, **, *p* < 0.01 compared with thymol-free culture.

The MICs of enrofloxacin and thymol against XS-91-4-1 were determined by the micro-dilution method. The MIC of enrofloxacin was 4 μg/mL and of thymol was 128 μg/mL. The result demonstrates that thymol had little inhibitory effect against *A. hydrophila*. As shown in Figure 2A, the influence of thymol on the growth of *A. hydrophila* was assessed by growth curve assays. As expected, sub-inhibitory concentrations ranging from 8 to 64 μg/mL had no impact on the growth of *A. hydrophila*, while the growth was clearly inhibited when co-cultured with thymol at the concentration of 128 μg/mL in 5 h. These results indicate that the growth of *A. hydrophila* is not impacted when treated with thymol at concentrations below 128 μg/mL.

### 3.2. Thymol Inhibits the Hemolytic Activity by Decreasing the Production of Aerolysin

To analyze the effect of sub-inhibitory concentrations of thymol on the hemolytic activity of *A. hydrophila* supernatants, hemolysis assay was performed. As shown in Figure 2B, thymol could inhibit the hemolytic activity of supernatant from *A. hydrophila* in a dose-dependent manner. The hemolytic activity decreased to 68.37%, 55.07%, 38.32%, and 13.96% when co-cultured with thymol at concentrations of 2, 4, 8, and 16 μg/mL, respectively. Compared with the drug-free group, the hemolytic activity was significant inhibited when co-cultured with thymol higher than 2 μg/mL.

**Figure 3:**
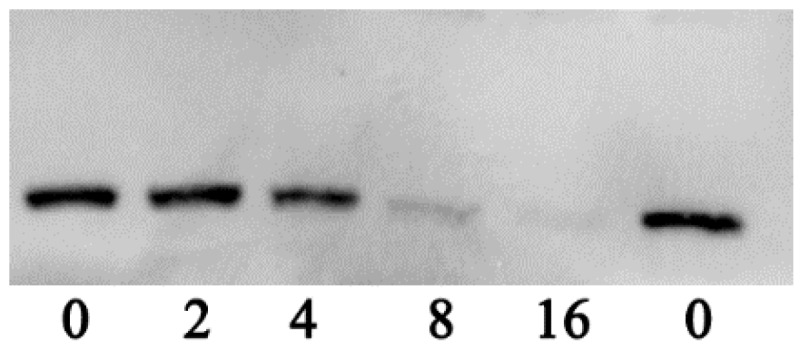
Western blot assay of aerolysin production in supernatants of *A. hydrophila* co-cultured with indicated concentrations of thymol.

To clarify whether the decrease of hemolytic activity was caused by the reduction of aerolysin in the supernatants when co-cultured with various concentrations of thymol, Western blot assay was performed. As expected, the production of aerolysin exhibited a similar manner as with the hemolysis assay, namely, the amount of aerolysin in the supernatants had a dose-dependent reduction when treated with thymol (Figure 3). Moreover, the immunoreactive aerolysin antigen was hardly detected in the supernatant treated with 8 and 16 μg/mL thymol (Figure 3). Taken together, thymol significantly reduced the hemolytic activity of supernatants of *A. hydrophila* by decreasing the production of aerolysin in the cultures at sub-inhibitory concentrations.

### 3.3. Thymol Reduces the Formation of Biofilm

**Figure 4:**
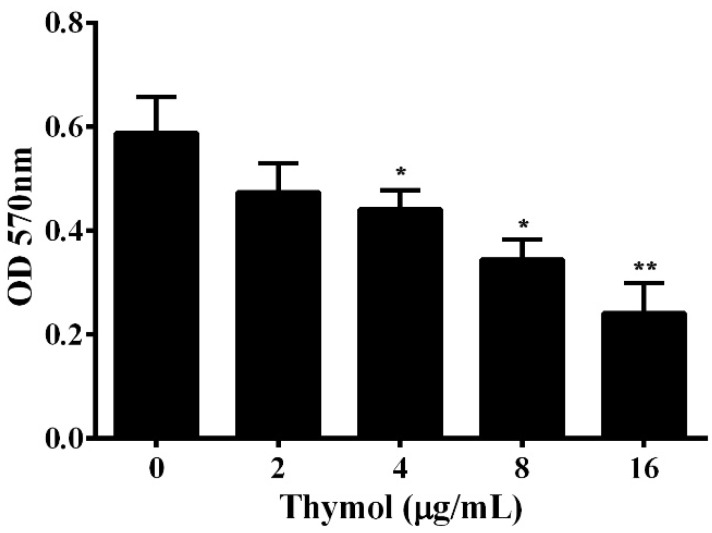
Influence of thymol on *A. hydrophila* biofilm formation. Biofilm formation assay was performed in a 96-well plate and the amount of biofilm was determined 24 h after thymol was added into the plate. The results are presented as the mean value ± SD (*n* = 3) (*, *p* < 0.05; **, *p* < 0.01).

Contamination from bacterial persistence and antibiotic resistance is due to the formation of biofilm, which protects the bacteria from being washed away [19]. According to previous studies, about 90% of bacteria can form biofilms [20]. Biofilm has been demonstrated to be involved in the antibiotic resistance and virulence of pathogenic *A. hydrophila* [21,22]. Thus, the impact of thymol on the formation of biofilm was determined in a 96-well plate. The results show that thymol could significantly reduce the biofilm formation of *A. hydrophila* in a dose-dependent manner (Figure 4). When treated with thymol at concentrations higher than 2 μg/mL, the biofilm was significantly decreased (Figure 4).

### 3.4. Real-Time PCR

**Figure 5:**
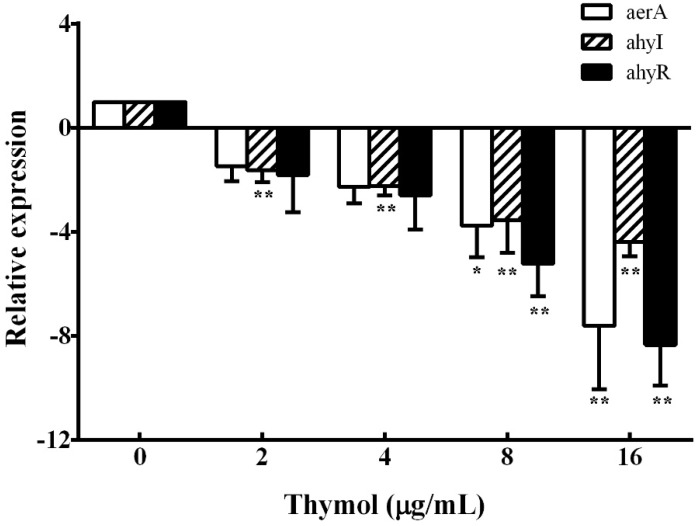
Relative expression of *aerA*, *ahyI*, and *ahyR* genes in *A. hydrophila* was determined by real-time PCR after treatment with indicated concentrations of thymol. All samples were analyzed in triplicate and data are shown as mean value ± SD. *, *p* < 0.05 and **, *p* < 0.01 when compared with the drug-free supernatants.

According to the results of hemolysis assay and biofilm formation assay, we found that thymol could inhibit the production of aerolysin and biofilm. These results indicate that thymol might reduce the hemolytic activity and biofilm formation of *A. hydrophila* by inhibiting the quorum sensing (QS) system. Thus, the transcription of aerolysin encoding gene *aerA*, QS signaling pathway regulator *ahyI*, and *ahyR* were studied. As shown in Figure 5, compared with the thymol-free group, the *aerA* gene was down-regulated 7.61-fold when treated with 16 μg/mL thymol. Similarly, *ahyI* and *ahyR* were also down-regulated 4.39- and 8.34-fold, respectively, when treated with 16 μg/mL thymol.

### 3.5. Thymol Protects A549 Cells from Cell Injury Induced by A. hydrophila

**Figure 6:**
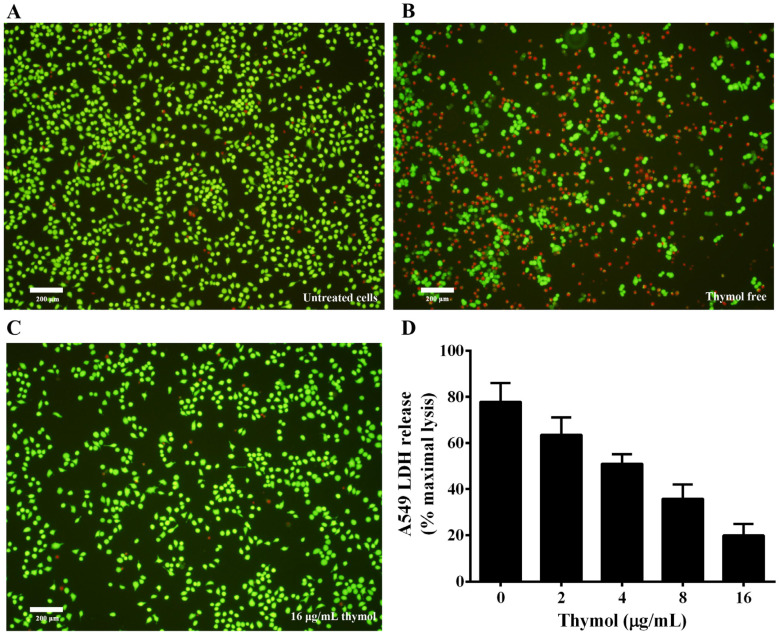
Thymol protects A549 cells against aerolysin-mediated cell injury. For live/dead assay, cells were stained with a LIVE/DEAD Viability/Cytotoxicity Kit and images were captured by a fluorescence microscope. A fluorescent-red dye stained dead cells, while a fluorescent-green dye stained live cells. For lactate dehydrogenase (LDH) assays, LDH release was detected by a Cytotoxicity Detection Kit. (**A**) untreated cells; (**B**) cells treated with bacterial supernatant without thymol; (**C**) cells treated with supernatant of *A. hydrophila* co-cultured with 16 μg/mL thymol; (**D**) LDH release of A549 cells when treated with supernatants co-cultured with indicated concentrations of thymol. All data are presented as mean value ± SD of three independent assays.

Previous studies have shown that aerolysin could form a pore resulting in cell death on target cells by binding to the glycosylphosphatidyl inositol (GPI)-anchored proteins of the target cell [23]. A number of mammalian cells have been reported to be sensitive to the toxin [23]. Thus, A549 cells were used to evaluate the protective effect of thymol against cell injury caused by aerolysin in *A. hydrophila* supernatant. Supernatants of *A. hydrophila* co-cultured with indicated concentrations of thymol were loaded into A549 cells, then cells were stained with a live/dead regent. As shown in Figure 6A, live cells were stained with green, while dead cells were stained red (Figure 6B). Dead cells significantly decreased when treated with supernatant plus 16 μg/mL thymol compared with cells treated with supernatant without thymol (Figure 6C). Furthermore, LDH release assay was performed to assess the cell viability when treated with supernatants of *A. hydrophila* plus different concentrations of thymol. As shown in Figure 6D, thymol could significantly decrease the cell injury induced by aerolysin in the supernatants from 2 to 16 μg/mL. Taken together, these results demonstrated that thymol could protect A549 cells from cell injury mediated by aerolysin in vitro.

### 3.6. Thymol Decreases the Mortality of Channel Catfish Infected with A. hydrophila

**Figure 7:**
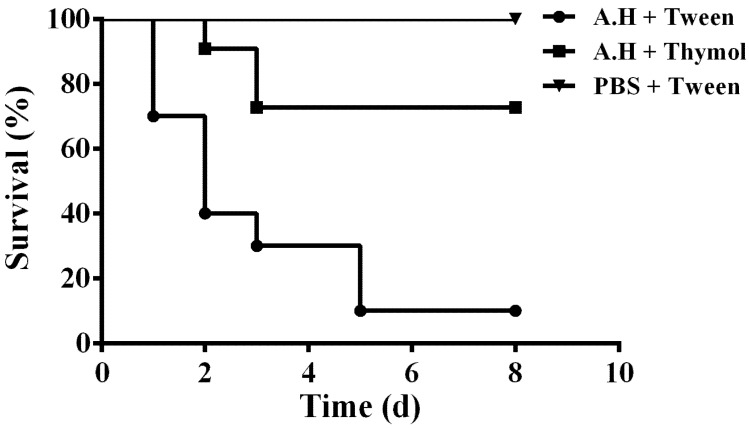
Thymol treatment increased the survival of channel catfish post infection. Infected channel catfish were administered with thymol or PBS, and the survival of the channel catfish was monitored for 8 days. A.H + Tween: group infected with *A. hydrophila* and treated with 10% Tween 80; A.H + Thymol: group infected with *A. hydrophila* and treated with thymol; PBS + Tween: group infected with PBS and treated with 10% Tween 80. The survival rate for the thymol-treated group was significantly increased compared to the positive control group when analyzed by the log-rank test (*p* < 0.0001 for the thymol-treated group).

The in vitro results demonstrated that thymol could significantly decrease the production of aerolysin, biofilm, and injury of A549 cells mediated by aerolysin in sub-inhibitory concentrations. Taken together, these results indicate that thymol has potential therapeutic effects against *A. hydrophila* infections. Thus, the infection models were established by infecting channel catfish with *A. hydrophila*. The channel catfish infected with *A. hydrophila* showed swelling around the fin and weight loss. Deaths were observed from 24 h post infection. As shown in Figure 7, the survival rate of infected fish following administration with thymol was 70%, while it was 10% for fish infected with *A. hydrophila* and administered with Tween 80. No deaths were observed in the group injected with sterile PBS (Figure 7). Taken together, thymol could significantly decrease the mortality of the channel catfish infected with *A. hydrophila*.

## 4. Discussion

The discovery of antibiotics has always been considered as one of the most relevant breakthroughs of the twentieth century. The introduction of antibiotics has revolutionized the treatments of infectious diseases caused by bacterial pathogens and contributed to the increase in life expectancy [24,25]. However, antimicrobial resistance has emerged due to the widespread use of antibiotics, and often leads to treatment failure [26]. Thus, anti-virulence strategies were devised by inhibiting pathogenicity or the ability to sustain an infection, instead of inhibiting or killing the bacteria [25]. In contrast with traditional antibiotics, targets of anti-virulence strategies are not essential for a bacteria’s life cycle, and less selective pressure will be provided. Therefore, agents generated by the strategy are less likely to develop antimicrobial resistance [27]. A number of targets have been clarified, such as the sulfur assimilation pathway, quorum sensing, toxins, adhesion, and biofilms [28,29].

*A. hydrophila* is an opportunistic pathogen distributed variously in the aquatic environment, and corresponds to several diseases in humans and terrestrial and aquatic animals [3]. High mortality caused by *A. hydrophila* in fish farming results in severe economic losses and threatens the healthy development of freshwater aquaculture in China. Treatment failure by traditional antibiotics has often been observed because of the spread of antimicrobial resistance. Moreover, aquatic systems provide the drivers of antimicrobial resistant genes, which might disseminate to clinical bacteria [30]. Therefore, control of resistant *A. hydrophila* strains is not only beneficial to fish farming but also to human health. Pore-forming toxins are one of the most common bacterial cytotoxic proteins and essential for the virulence of a number of bacterial pathogens [31], and have been recognized as targets in developing novel drugs. Thus, aerolysin, a well-studied pore-forming toxin, was employed as a target in screening drugs against *A. hydrophila* infections from natural compounds. Previous studies have demonstrated that aerolysin is essential for the virulence of *A. hydrophila* [12,32]. Our previous study demonstrated that magnolol could significantly reduce the pathogenicity of *A. hydrophila* by inhibiting the transcription of the *aerA* gene. However, there was no significant influence on biofilm formation and the QS signaling pathway. As is known, the QS signaling pathway directly relates to the pathogenesis of *A. hydrophila*. Thus, the QS mechanism has been identified as a novel target in developing antibacterial drugs [18]. In the present study, we found that 4 μg/mL thymol could significantly reduce the hemolytic activity of *A. hydrophila* supernatant to 55.07 ± 4.85% compared with the thymol-free supernatant (Figure 2B). At the same concentration, thymol could significantly reduce the quantity of biofilm when measured by OD_570 nm_. According to the results of growth curves (Figure 2A), our experimental concentrations of thymol showed little effect on the growth of *A. hydrophila*. Several virulence factors, including aerolysin and biofilm formation, are partially attributed to the QS mechanism, indicating that thymol might inhibit the QS system. Consequently, QS-related genes were selected to investigate the influence of thymol on the QS system. As expected, we found that thymol could increase the survival rate of channel catfish infected with *A. hydrophila* by inhibiting the QS signaling pathway and aerolysin and biofilm production. Previous research demonstrated that rosmarinic acid could reduce the pathogenicity of *A. hydrophila* both in vitro and in vivo by inhibiting the QS signaling pathway and production of aerolysin and biofilm [33]. Although rosmarinic acid could protect zebrafish from *A. hydrophila* infection, higher concentrations of rosmarinic acid exhibited toxicity to this species. The safety of this agent might limit its application in fish farming. As described previously, thymol at a dosage of 100 mg/kg could significantly increase the survival rate of grass carp post infection of *A. hydrophila* [34]. The findings indicated that thymol is safe for fish in doses lower than 100 mg/kg. Studies published by Morselli et al. [34] and Cunha et al. [35] demonstrated that thymol could decrease *A. hydrophila*-mediated mortality in grass carp and *Rhamdia quelen*. However, the influence of thymol on *A. hydrophila* has not been well studied. Although thymol had a sight inhibitory effect against *A. hydrophila* in vitro according to our MIC assay, the concentration of thymol could not reach 128 μg/mL in plasma. Thus, thymol could not protect fish from *A. hydrophila* infections according to the MIC value. Previous research showed that thymol and some other phytochemicals had synergic effects against fish pathogenic bacteria by checkerboard assay [36]. Moreover, the inhibitory effects of thymol on *A. hydrophila* hemolysis and biofilm formation have been demonstrated. Although the MIC reported by G. Bandeira et al. was lower than that reported in this study [36], the hemolytic and biofilm formation inhibitory concentration was higher than in this study. As shown in Figure 2B, the hemolytic inhibitory concentration was about 2.5 times lower than that reported by G. Bandeira et al. [35]. Different cultivation time and strains might result in different hemolytic and biofilm inhibitory activity. In addition, the influence of thymol on the virulence factor and QS signaling pathway was systematically investigated, which clarified the mechanism of thymol against *A. hydrophila*.

Thymol has been reported to exhibit several biological activities that could reduce pathogenicity of a number of bacterial pathogens. Yuan and Yuk [37] demonstrated that thymol could reduce motility, biofilm formation, and efflux pump activity of *Escherichia. coli* O157:H7 with an induction of antimicrobial resistance. Zhang et al. [38] showed that thymol could protect mice from *Salmonella typhimurium*-induced infection by inhibiting its Type III secretion system. Yin et al. [39] found that supplementation with a BEOs diet could reduce the virulence factor of *Clostridium perfringens* and the resulting mortality of chicken. These findings indicate that thymol could be chosen as a novel drug against bacterial pathogens.

## Figures and Tables

**Table 1:** Primer pairs used for RT-PCR.

Primer	Sequence (5′-3′)	PCR Amplicon (bp)	Reference
*aerA*-F	TCTACCACCACCTCCCTGTC	218	[3]
*aerA*-R	GACGAAGGTGTGGTTCCAGT		
*ahyR*-F	TTTACGGGTGACCTGATTGAG	206	[18]
*ahyR*-R	CCTGGATGTCCAACTACATCTT		
*ahyI*-F	GTCAGCTCCCACACGTCGTT	202	this study
*ahyI*-R	GGGATGTGGAATCCCACCGT		
*16S rRNA*-F	TAATACCGCATACGCCCTAC	164	[3]
*16S rRNA*-R	ACCGTGTCTCAGTTCCAGTG		
